# Global trends of peripheral immune tolerance research: a bibliometric and visualization analysis

**DOI:** 10.3389/fimmu.2026.1730575

**Published:** 2026-02-05

**Authors:** Shixiao Kong, Chen Lin, Yuting Shen, Jun Chen

**Affiliations:** 1 School of Chemistry and Chemical Engineering, Wuhan University of Science and Technology, Wuhan, China; 2 School of Nursing, Shaanxi University of Chinese Medicine, Xianyang, China

**Keywords:** bibliometric analysis, immune regulation, peripheral immune tolerance, regulatory T cells (Tregs), research hotspots, research trends

## Abstract

**Background:**

Peripheral immune tolerance is a key mechanism for maintaining immune homeostasis and preventing autoimmune responses. Although numerous studies have explored its molecular and cellular basis, a comprehensive and systematic overview of the field is still lacking. This study aims to elucidate the global research landscape of peripheral immune tolerance through bibliometric analysis.

**Methods:**

Publications related to peripheral immune tolerance from 1989 to 2025 were retrieved from the Web of Science Core Collection and Scopus databases. After removing duplicates, a total of 3,098 papers were included. CiteSpace, VOSviewer, RStudio Biblioshiny, and OriginPro were used for systematic analysis, allowing effective identification of research trends and emerging topics.

**Results:**

Since the 1990s, the annual number of publications on peripheral immune tolerance has steadily increased, peaking in 2008 before showing a slight decline. High-frequency keywords included autoimmunity, tolerance, and regulatory T cells. The United States, Germany, China, and Japan were the leading contributors, with Harvard University ranking first in both publication volume and citation frequency. Highly cited landmark papers by Sakaguchi, Freeman, and Curiel laid the theoretical foundation for the field. The Journal of Immunology had the highest publication count, while Nature Immunology showed the greatest impact.

**Conclusion:**

This study provides the first comprehensive bibliometric analysis of global research on peripheral immune tolerance, revealing the evolution of research themes over the past three decades and forecasting future trends. The findings offer valuable data support and insights for advancing research in this field.

Systematic review registration:

## 1 Introduction

Immune tolerance is a crucial physiological mechanism by which the immune system distinguishes self from non-self antigens, thereby preventing excessive or autoimmune responses. Among its various forms, peripheral immune tolerance plays a pivotal role in maintaining immune homeostasis (1, 2). In 1990, Corbel and colleagues (3) demonstrated that peripheral immune tolerance could be established against exogenous antigens in the peripheral tissues of chicken embryos, providing direct evidence for the existence of peripheral immune tolerance.

Over the past 3 decades, remarkable progress has been made in elucidating the molecular mechanisms underlying peripheral immune tolerance. This mechanism has shown broad applicability in organ transplantation (4), the treatment of autoimmune diseases (5), allergen desensitization (6), and tumor immunosuppression (7). When the function of peripheral immune tolerance is impaired, it can lead to the development of autoimmune disorders such as rheumatoid arthritis, systemic lupus erythematosus, and type 1 diabetes (8). Therefore, a comprehensive understanding of the regulatory mechanisms and pathological roles of peripheral immune tolerance is of great significance for both basic immunological research and clinical translation.

However, as research directions within this field have diversified, the overall landscape, knowledge structure, and emerging hotspots of peripheral immune tolerance have become increasingly complex. The absence of systematic quantitative analyses has hindered accurate identification of research trends, key contributors, and potential frontiers. Consequently, a global bibliometric assessment of this field is warranted to elucidate its academic evolution and future development trajectories.

Bibliometrics is a well-established and efficient tool for literature review and thematic mining, capable of quantitatively revealing the overall structure and dynamic evolution of a research field (9). Applying bibliometric methods to the study of peripheral immune tolerance facilitates a comprehensive depiction of its publication trends, core scholars and institutions, international collaboration patterns, and thematic evolution pathways. Such analysis not only uncovers theknowledge base and research hotspots of the discipline but also provides valuable insights and guidance for future studies focusing on mechanistic exploration, clinical translation, and cross-disciplinary applications. Based on this rationale, the present study systematically retrieved and integrated literature related to peripheral immune tolerance from the Web of Science Core Collection and Scopus databases. Using VOSviewer, CiteSpace, RStudio Biblioshiny, and OriginPro, a comprehensive bibliometric and visualization analysis was conducted to delineate the global research landscape, knowledge structure, emerging topics, and development trends of this field from 1989 to 2025.

## 2 Materials and methods

### 2.1 Data retrieval

This bibliometric study was conducted in accordance with the Guidance List for repOrting Bibliometric AnaLyses (GLOBAL). We followed all relevant checklist items to ensure transparent, reproducible, and methodologically rigorous reporting. Specifically, we: (i) clearly defined the research objectives and analytical framework; (ii) provided full details of data sources, database selection rationale, and search strategies; (iii) reported inclusion and exclusion criteria and documented data cleaning procedures; (iv) described all bibliometric tools and analytical parameters used; (v) ensured reproducibility by specifying software versions and settings; (vi) presented visualization outputs in accordance with recommended standards; and (vii) acknowledged methodological constraints and potential biases.

The data for this study were retrieved and collected from the Web of Science Core Collection (WoSCC) and Scopus databases, both of which are recognized as authoritative and comprehensive sources of scholarly information with high data integrity (10).

PubMed was excluded from this study because its records lack several key metadata fields that are essential for conducting standardized bibliometric analyses. Unlike the WoSCC, PubMed does not provide citation counts, reference lists, or other citation-linking information. Moreover, it is difficult to reliably restrict searches to “articles” and “reviews,” as PubMed uses its own unique categorization system that includes a substantial number of clinical-trial–related records. In practice, PubMed entries may also lack affiliation and country information, largely due to the fact that mainstream bibliometric tools are optimized for the data structures of WoSCC and Scopus rather than PubMed. To prevent analytical inconsistencies and bias arising from incomplete metadata, we used only Scopus and WoSCC, both of which offer more comprehensive and software-compatible datasets for bibliometric research.

In WoSCC, the search period was set from January 1, 1900, to October 1, 2025, and the document types were limited to articles and reviews. The search strategy was: TS = (“peripheral immune tolerance” OR “peripheral tolerance”) NOT TS = (“central tolerance” OR “central immune tolerance”), which yielded 2,006 publications.

In Scopus, the search query was: TITLE-ABS-KEY(“peripheral immune tolerance” OR “peripheral tolerance”) AND NOT TITLE-ABS-KEY(“central tolerance” OR “central immune tolerance”), resulting in 2,762 publications.

This dual-database approach ensured a precise focus on peripheral immune tolerance while minimizing “noise” associated with central (thymic) immune tolerance. To maintain consistency, identical settings and a uniform time range were applied in both databases. All retrieved records were then merged and deduplicated using RStudio Biblioshiny, resulting in 1,670 duplicated entries. In bibliometric analysis, data accuracy and reproducibility are of critical importance (11–13). Therefore, the entire retrieval, cleaning, and merging process was repeated three times to ensure there were no errors or inconsistencies in data collection and integration.

**Figure 1:**
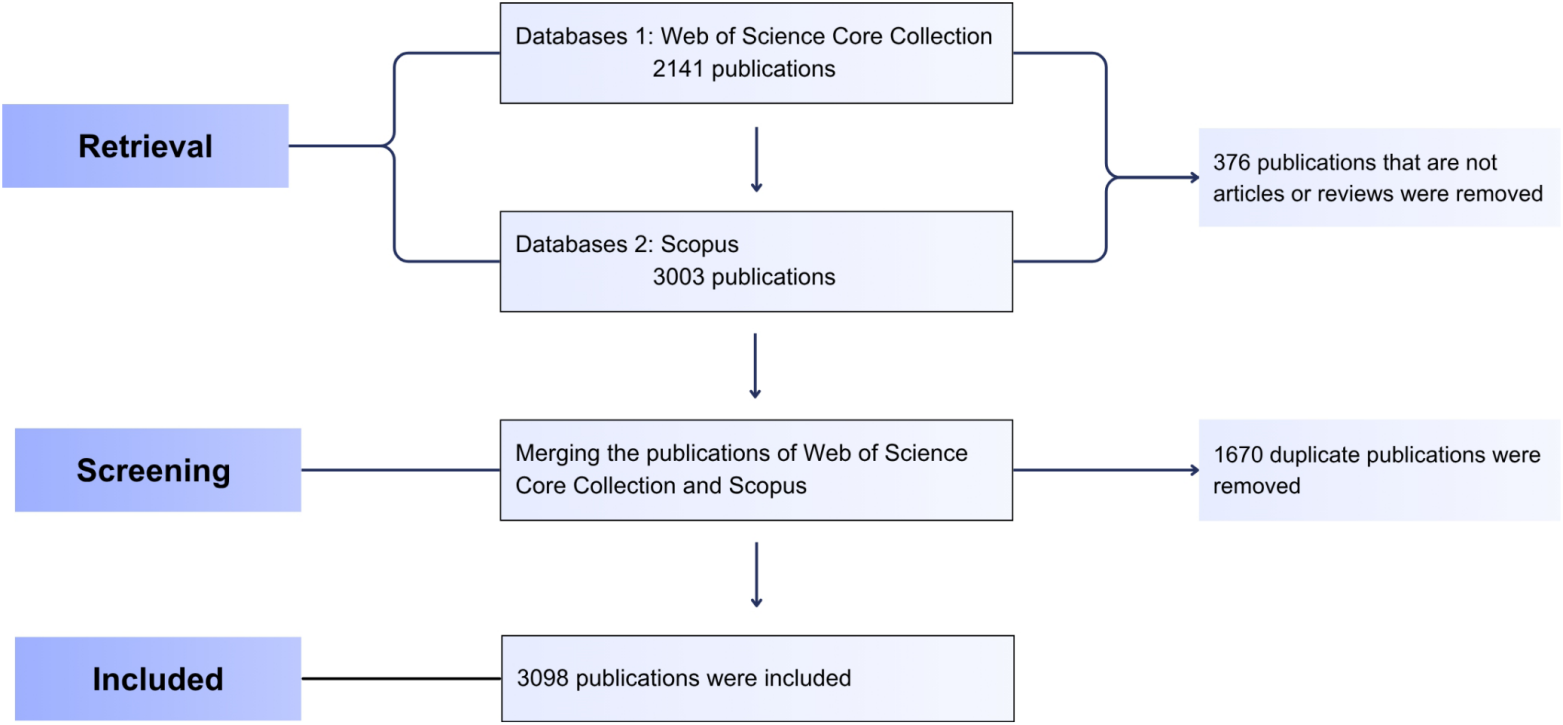
Flowchart of the retrieval and screening process.

The overall retrieval process is illustrated in **Figure 1**.

### 2.2 Data analysis

Bibliometrics involves the use of statistical methods to analyze publication information and its associated metadata, including abstracts, keywords, and citations (14). In this study, a combination of software tools was employed for bibliometric visualization, including VOSviewer 1.6.20, CiteSpace 6.4.R1, RStudio Biblioshiny 5.0, and OriginPro 2024.

VOSviewer is a free software tool designed to construct and clearly visualize large-scale bibliometric networks (15). The retrieved data were imported into VOSviewer to generate co-occurrence networks. CiteSpace and RStudio Biblioshiny were used for burst detection and hotspot evolution analysis, enabling the identification of emerging research fronts and thematic trends. Finally, OriginPro was applied to create various statistical charts and plots, supporting further quantitative visualization.

### 2.3 Ethics exemption statement

This study is a bibliometric analysis based solely on previously published academic literature. All data were obtained from publicly accessible scholarly databases and consist exclusively of secondary metadata (including titles, authors, affiliations, abstracts, keywords, and citation information). No human participants, animals, clinical interventions, or identifiable personal information were involved, nor does the study evaluate individual authors. As the research relies entirely on publicly available data and involves no ethical concerns related to human or animal subjects, formal approval from an ethics committee was deemed unnecessary.

## 3 Results

### 3.1 Publishing trend

**Figure 2:**
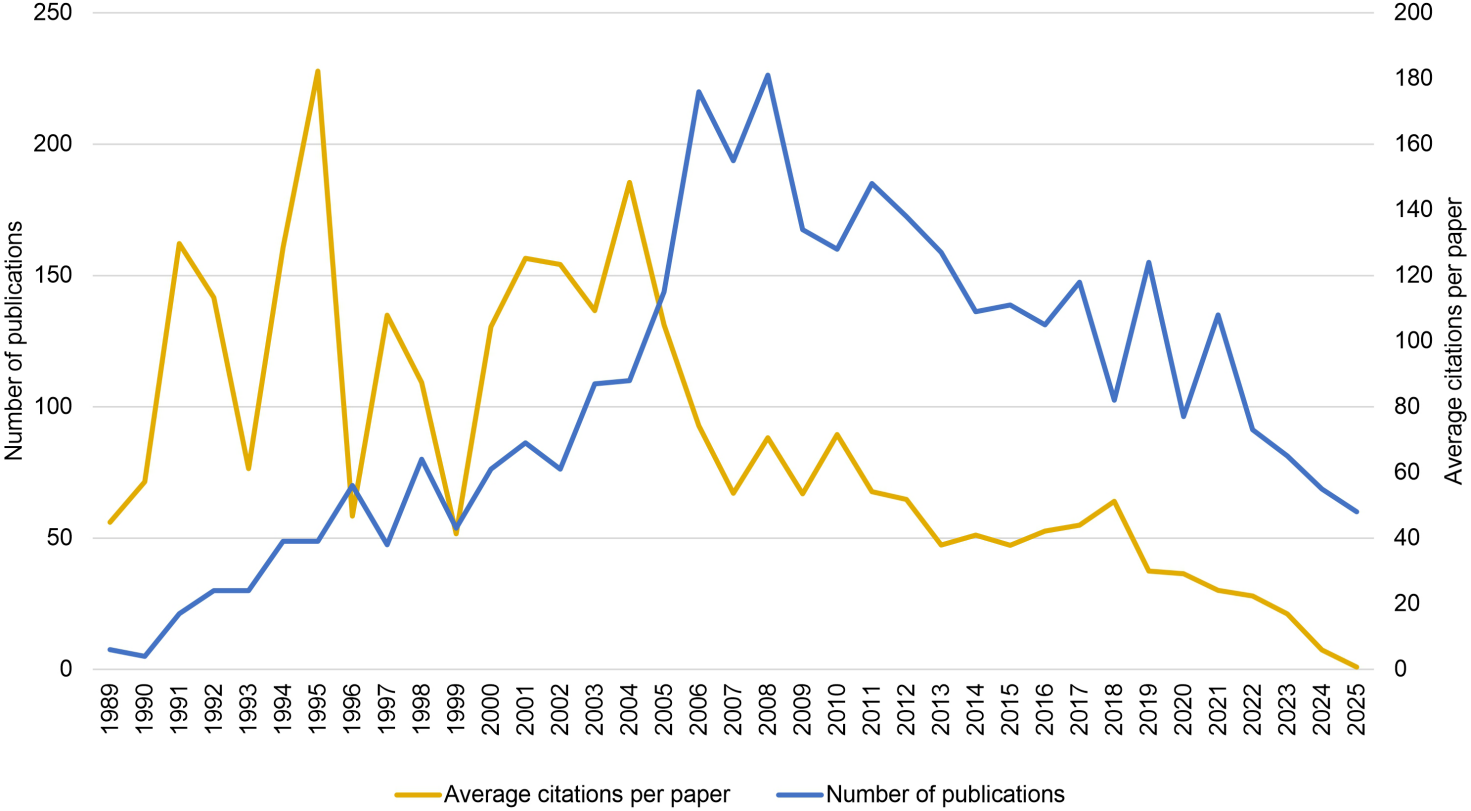
The publication trends and citation counts of research on peripheral immune tolerance, based on the merged data from the WOSCC and Scopus databases.

**Figure 2** illustrates the temporal evolution of the annual number of publications and the average citation frequency in the field of peripheral immune tolerance from 1989 to 2025. The annual publication output began to increase gradually in the early 1990s and reached a peak in 2008 with 181 publications. Although it fluctuated thereafter, an overall declining trend was observed, decreasing to 48 publications by 2025. In contrast, the average citations per paper peaked earlier, reaching 227.79 citations per paper in 1995, followed by fluctuations and a general downward trend. Since 2011, the value has remained consistently below 70 citations per paper, dropping to 7.44 in 2024. However, given the inherent time lag in citation accumulation, the average citation frequency is expected to increase after 2025.

### 3.2 Research hotspots

**Figure 3:**
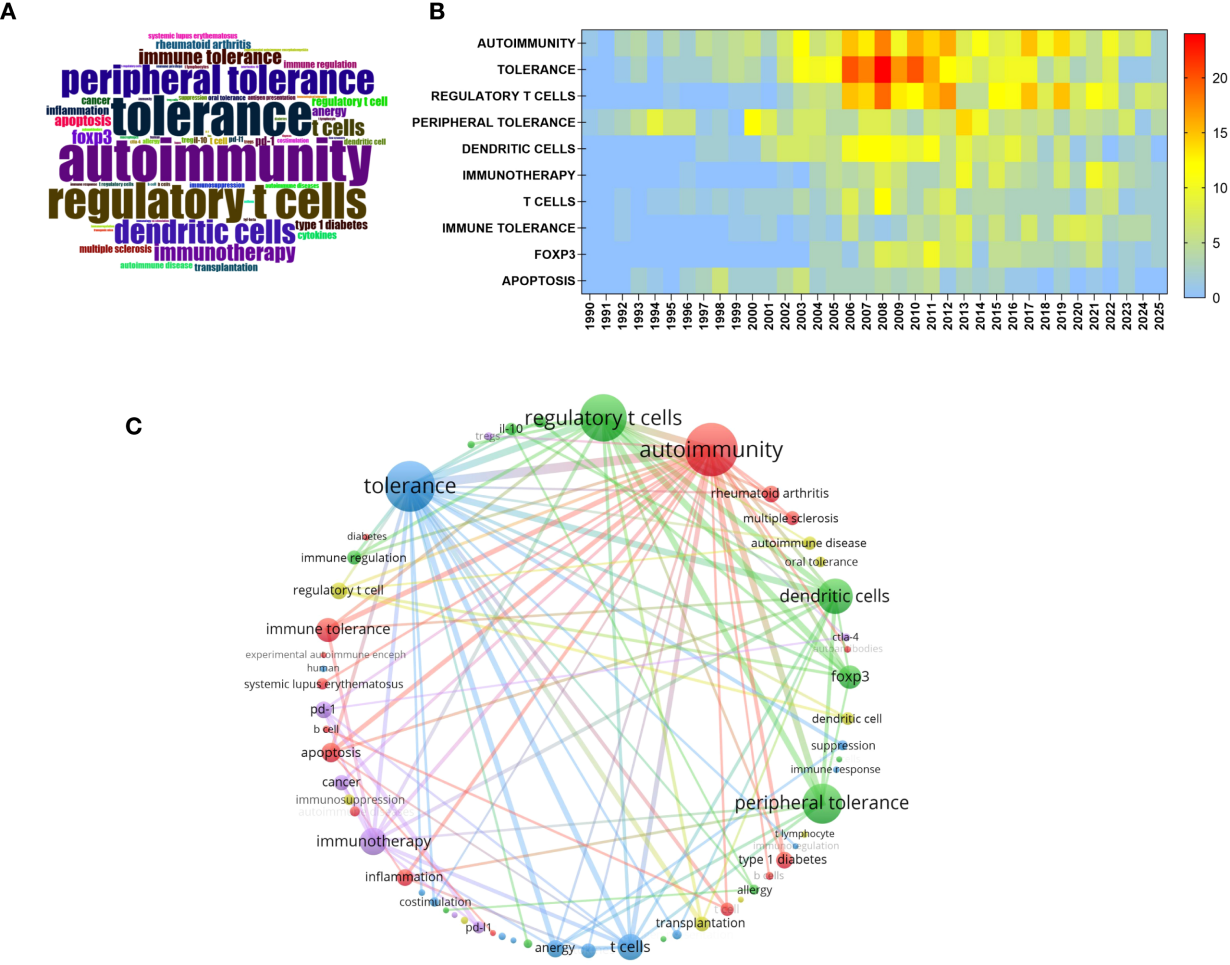
Hotspot keywords of research on peripheral immune tolerance. **(A)** Word cloud of high-frequency keywords. **(B)** Heat map of the top 10 most frequent keywords. **(C)** Keyword co-occurrence network for keywords appearing more than 135 times.

We first performed a keyword analysis using RStudio Biblioshiny and VOSviewer. **Figure 3** presents the results of the keyword visualization analysis in the field of peripheral immune tolerance. **Figure 3A** shows the keyword co-occurrence word cloud, in which the font size of each term is proportional to its frequency of appearance. The results indicate that “autoimmunity,” “tolerance,” “regulatory T cells,” “peripheral tolerance,” and “dendritic cells” were the most frequently occurring keywords. **Figure 3B** displays the temporal heat map of high-frequency keywords, where the color gradient from blue to red represents increasing frequency. The results reveal that since 2005, keywords such as “autoimmunity,” “tolerance,” and “regulatory T cells” have appeared frequently and continued to gain prominence, whereas emerging keywords such as “immunotherapy” and “Forkhead box P3 (FOXP3)” have gradually become more visible in recent years. The FOXP3 gene encodes a key transcription factor predominantly expressed in regulatory T cells (Tregs), underscoring its crucial role in the regulatory mechanisms of peripheral immune tolerance. This analysis provides a more intuitive and precise understanding of the key research themes and their evolving trends within this field.

**Figure 3C** illustrates the keyword co-occurrence network of the peripheral immune tolerance field constructed using VOSviewer. The overall network exhibits a multicluster structure. The red cluster primarily contains the keywords “autoimmunity” and “regulatory T cells,” while the blue cluster centers around “tolerance” and “immune tolerance.” The green cluster is organized around “dendritic cells” and “peripheral tolerance,” focusing on antigen presentation and peripheral immune regulation. High-frequency keywords include “autoimmunity” (287 occurrences), “tolerance” (266 occurrences), “regulatory T cells” (240 occurrences), and “peripheral tolerance” (194 occurrences). These keywords are highly interconnected through multiple co-occurrence links, collectively forming the core thematic framework of research within this field.

**Figure 4:**
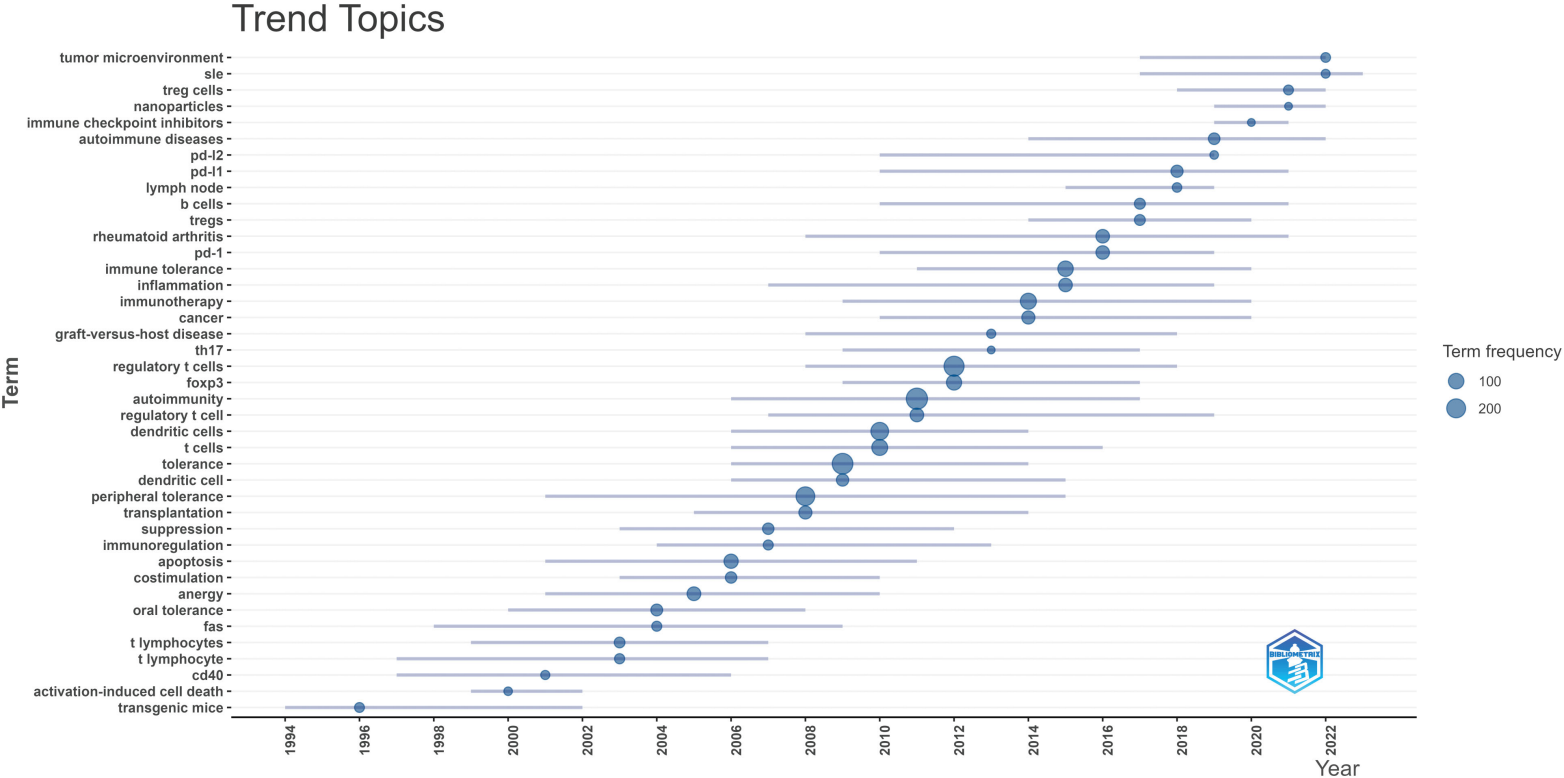
Trend topics in the field of peripheral immune tolerance based on all keywords (author keywords and keywords plus).

The keyword trend analysis reveals a clear evolution of research themes across different periods. It can be identified from **Figure 4** that in the early stage (1990s–2005), studies predominantly focused on fundamental immunological mechanisms, with frequently occurring terms such as “transgenic mice”, “activation-induced cell death”, “T lymphocyte”, “CD40”, “oral tolerance”, “Fas”, and “peripheral tolerance”. These keywords highlight strong interest in immune cell function, tolerance induction, and apoptotic processes, reflecting a mechanism-oriented research pattern. During the middle stage (2006–2015), research attention expanded toward immune regulatory networks and disease-related mechanisms. Representative keywords include “dendritic cells”, “regulatory T cells”, “FOXP3”, “tolerance”, “immune tolerance”, “inflammation”, “transplantation”, “graft-versus-host disease”, and “rheumatoid arthritis”, indicating a shift from basic immunology to autoimmune disease models and immune regulatory pathways. The emergence of terms such as “PD-1” and “TH17” further signals the rise of new research directions concerning immune checkpoints and inflammatory cell subsets. In the recent stage (2016–2025), research themes became increasingly clinical and therapy-oriented, characterized by high-frequency terms such as “immune checkpoint inhibitors”, “immunotherapy”, “autoimmune diseases”, “SLE”, “Treg cells”, “nanoparticles”, and “tumor microenvironment”. These trends suggest that immunotherapy, autoimmune disease progression mechanisms, and tumor immune microenvironment have become central hotspots in the field.

### 3.3 Analysis of geographical distribution

**Figure 5:**
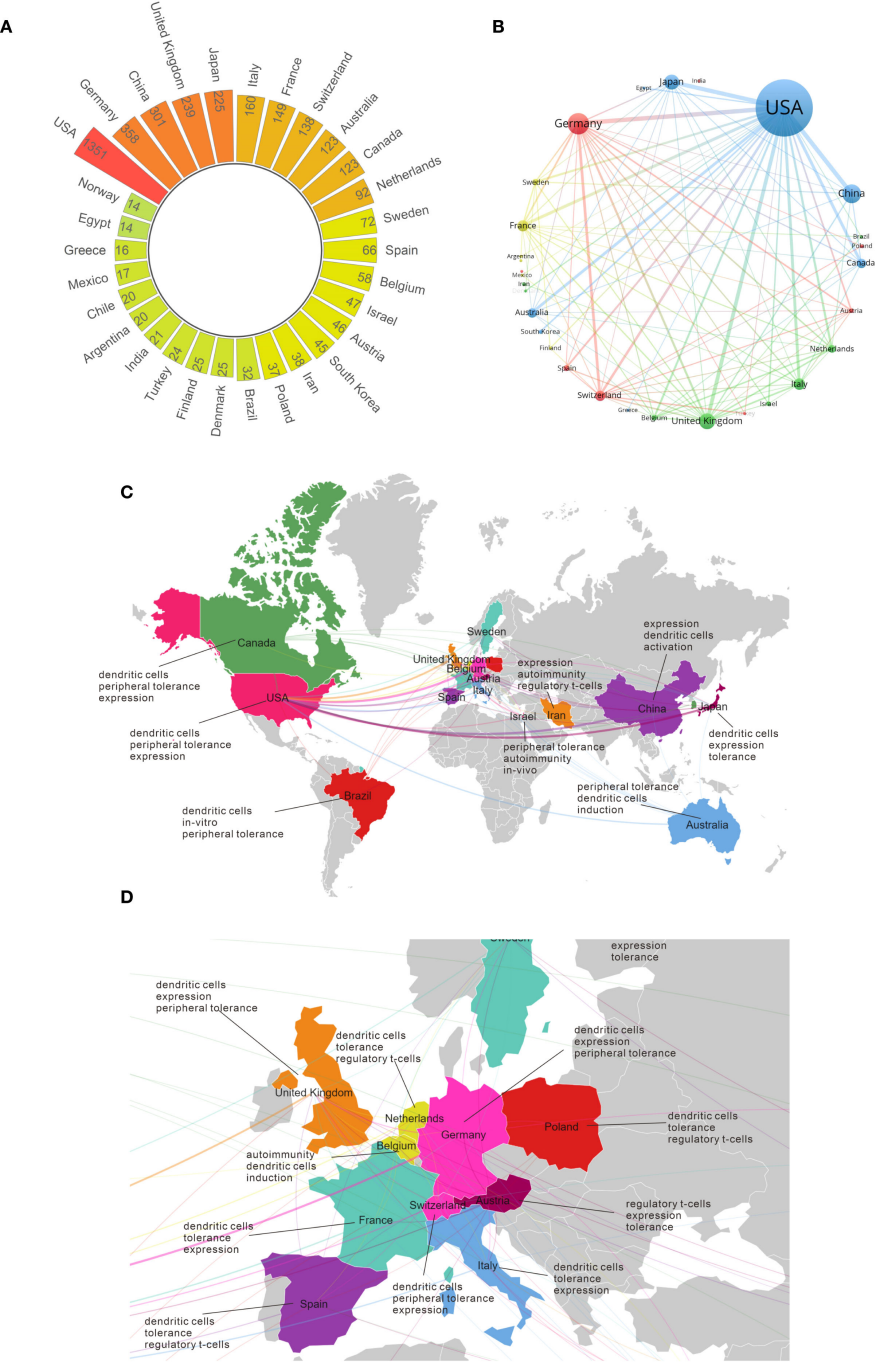
National publication analysis of research on peripheral immune tolerance. **(A)** Radial bar chart of the top 30 countries by publication output. **(B)** Country collaboration network of countries with more than 15 publications. **(C)** Geographical collaboration network of the top 20 most productive countries and their associated high-frequency research keywords. **(D)** Enlarged view of the European region.

We then focused on the geographical distribution of publications to identify the major contributing countries in the field of peripheral immune tolerance. **Figure 5** presents the global publication distribution and international collaboration network in this research area. **Figure 5A** shows that the United States ranks first by a large margin, with 1,351 publications, followed by Germany (358), China (301), the United Kingdom (239), and Japan (225). **Figure 5B** depicts the country collaboration network, indicating that the United States serves as the central collaboration hub in this field, maintaining close research partnerships with China, Japan, Germany, and multiple European countries. The geographic keyword co-occurrence maps in **Figures 5C, D** provide further insight into regional research emphases. **Figure 5C** further displays the global geographic distribution of research themes. The United States and Canada are mainly associated with keywords such as “dendritic cells”, “peripheral tolerance”, and “expression”. China and Japan also show frequent linkage to “dendritic cells”, with additional emphasis on “activation” and “tolerance”. Australia presents co-occurrence patterns involving “peripheral tolerance”, “dendritic cells”, and “induction”. Several European and Middle Eastern countries, including the United Kingdom, Belgium, Austria, Italy, and Iran, share common terms such as “tolerance”, “autoimmunity”, and “regulatory T-cells”. **Figure 5D** provides a focused view of Europe, where Germany, the Netherlands, Belgium, Switzerland, Austria, and Italy form a dense regional cluster characterized by recurring terms such as “dendritic cells”, “tolerance”, and “regulatory T-cells”. Overall, the European map indicates a relatively concentrated distribution of research themes related to dendritic cell biology and peripheral immune tolerance.

### 3.4 Analysis of geographical distribution

Through the use of various software platforms, we extracted and organized the primary information regarding authors and institutions, and the resulting data were compiled into **Tables 1**–**3**. TSL refers to total link strength, and the dash indicates that the data could not be verified.

**Table 1:** The top 10 institutions by publication count.

Rank	Institution	Publications	Citations	TLS
1	Harvard University	90	10768	92
2	Johannes Gutenberg University Mainz	41	2220	50
3	University of Pittsburgh	38	3441	26
4	University of Pennsylvania	35	3370	27
5	Harvard Medical School	34	2059	35
6	Brigham and Women’s Hospital	32	6372	43
7	Karolinska Institutet	30	1844	43
8	University of Washington	30	4538	33
9	The Scripps Research Institute	29	3205	9
10	University of California, San Francisco	28	6184	25

**Table 2:** Thematic evolution of research keywords among the top institutions (2006–2025).

Research Time keywords Institution	2006-2012	2013-2019	2020-2025
Harvard University	Tolerance; Tregs; PD-1; PD-L1	Dendritic cells; Tregs; Peripheral Tolerance; Autoimmunity	Dendritic cells; Tregs; Expression
Johannes Gutenberg University Mainz	Dendritic cells; Tregs; Tolerance	Dendritic cells; Tregs; Tolerance;	Tregs; FOXP3 expression
University of Pittsburgh	Tolerance; Tregs; Transplantation	Tregs; Transplantation	Dendritic cells; Tregs; Expression
University of Pennsylvania	Autoimmune-disease; Dendritic cells; SLE	Dendritic cells; Breast cancer; Tregs; NFkappa-B	FOXP3; Peripheral Tolerance
Harvard Medical School	Responses; T cell; TIM-3;	Autoimmunity; Tolerance; Cancer	SLE; Autoimmunity; Tregs

**Table 3:** The top 10 authors by publication count.

Rank	Author	Publications	Citations	TLS	Institution	Active year
1	Roncarolo, Maria Grazia	23	9801	35	San Raffaele Scientific Institute	1997-2023
2	Azuma, Miyuki	21	4606	49	Tokyo Medical and Dental University	1995-2015
3	Akdiş, Cezmi A A	20	4605	25	Swiss Institute of Allergy and Asthma Research	1996-2013
4	Akdiş, Mübeccel	20	4647	27	Swiss Institute of Allergy and Asthma Research	1996-2013
5	Stein-Streilein, Joan	20	1203	6	Harvard Medical School	2000-2010
6	Weiner, Howard L	18	6548	29	Brigham and Women’s Hospital	1991-2010
7	Bacchetta, Rosa	17	3958	17	Stanford University School of Medicine	2001-2024
8	Jonuleit, Helmut	17	1389	37	University of Mainz	2002-2016
9	Yagita, Hideki Y	16	2972	32	——	1995-2005
10	Sherman, Linda A	15	1246	18	The Scripps Research Institute	1991-2017

As shown in **Table 1**, Harvard University is the leading institution, with 90 publications, the highest citation count (10,768 citations), and the strongest TSL (16), underscoring its central scientific influence. Other major contributors include Johannes Gutenberg University Mainz (41 publications) and the University of Pittsburgh (38 publications), which show stable research output and strong collaborative activity. Several institutions—including Brigham and Women’s Hospital and the University of California, San Francisco—produce fewer papers but exhibit exceptionally high citation frequencies, indicating that their research outputs have a disproportionately large scientific impact within the field.

**Table 2** further reveals how the research priorities of leading institutions have evolved over the past 20 years. From 2006–2012, most institutions concentrated on classical tolerance-related mechanisms. Harvard University emphasized “tolerance,” “Tregs,” and the PD-1/PD-L1 pathway, reflecting early mechanistic studies on co-inhibitory signaling. Johannes Gutenberg University Mainz and the University of Pittsburgh focused on “dendritic cells,” “Tregs,” and “transplantation,” highlighting the importance of antigen-presenting cells and graft tolerance research during this period. The University of Pennsylvania showed strong interest in autoimmune diseases such as SLE, along with dendritic cell biology.

During 2013–2019, all institutions demonstrated a clear shift toward immune regulation in disease contexts. Harvard University, the University of Pittsburgh, and Mainz intensified their work on “dendritic cells,” “Tregs,” and “peripheral tolerance,” indicating a transition from pathway-centered research to integrated models of immune regulation. At the same time, keywords such as “breast cancer,” “NF-κB,” and “autoimmunity” became prominent in the output of the University of Pennsylvania and Harvard Medical School, reflecting the increasing relevance of peripheral immune tolerance mechanisms in tumor immunology and chronic inflammatory disorders.

In the most recent period (2020–2025), emerging research hotspots became evident. Several institutions—including Harvard University, Mainz, and Pittsburgh—focused heavily on “FOXP3 expression,” “Treg expression,” and the transcriptional regulation of regulatory T cells, aligning with the broader shift toward precision immunology and Treg-targeted therapies. The University of Pennsylvania emphasized “FOXP3” and “peripheral tolerance,” while Harvard Medical School’s work centered on “SLE,” “autoimmunity,” and “Tregs,” indicating growing attention to disease-driven dysregulation of peripheral immune tolerance.

**Table 3** summarizes the top 10 most productive authors in peripheral immune tolerance research. Roncarolo Maria Grazia ranks first with 23 publications and 9,801 citations, reflecting her leading influence in Treg biology research. Azuma Miyuki and Cezmi A. Akdis follow closely, each contributing over 20 publications with high citation counts and strong collaboration strength. Other prominent authors, including Mübeccel Akdis, Howard L. Weiner, and Rosa Bacchetta, also demonstrate substantial impact through high citation frequencies and active participation in collaborative networks. Together, these scholars constitute the core author group shaping the development of peripheral immune tolerance over the past decades.

### 3.5 Analysis of cited publications and references

**Figure 6:**
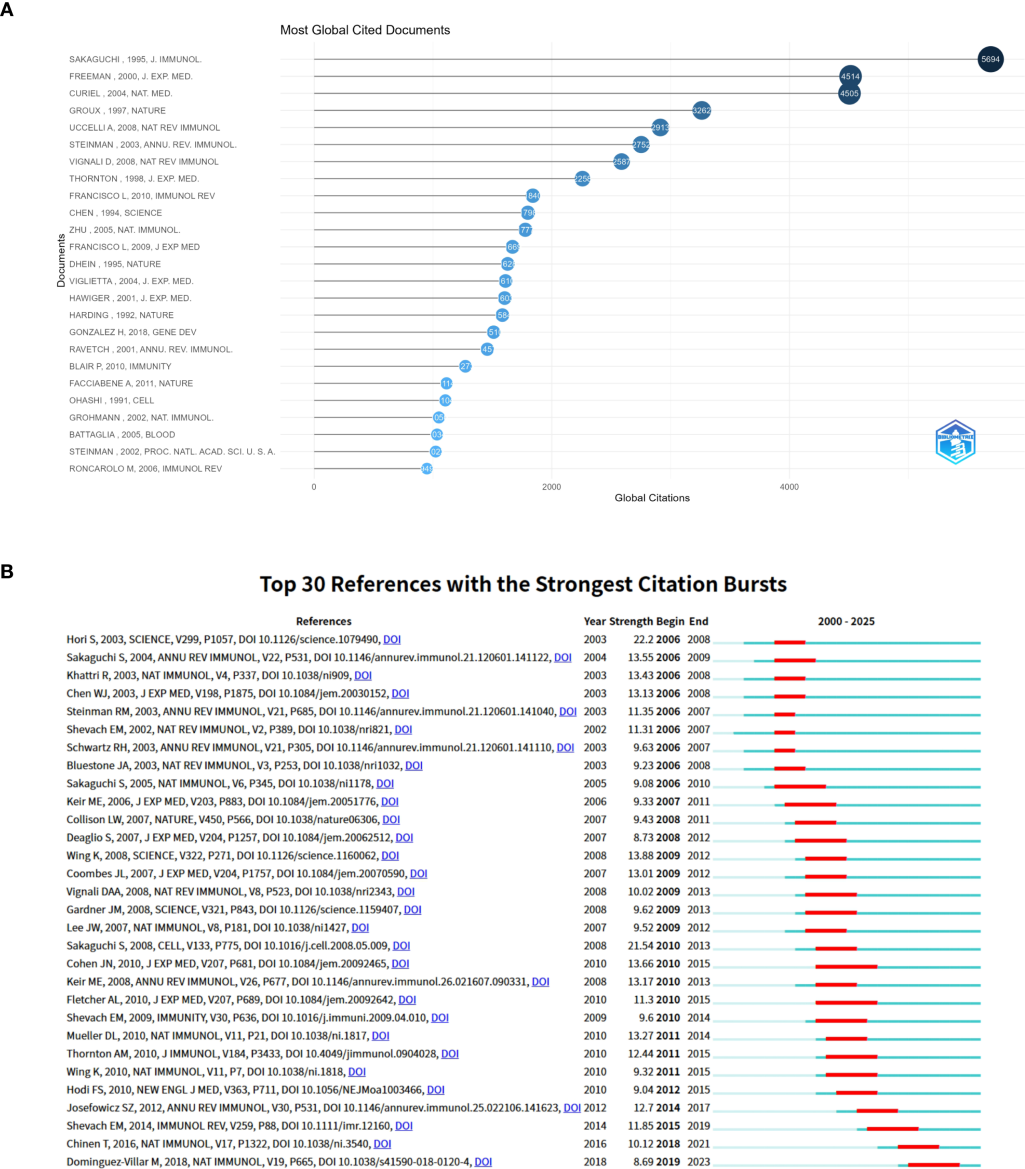
Citation analysis of publications of research on peripheral immune tolerance. **(A)** Top 25 most globally cited publications. **(B)** Top 30 references with the strongest citation bursts.

In bibliometrics, citation analysis is also an essential component, as it helps readers quickly identify high-impact publications within a specific research field. We conducted an analysis of co-cited and highly cited publications using CiteSpace and RStudio Biblioshiny. **Figure 6A** presents the most globally cited publications in the field of peripheral immune tolerance. The paper by Sakaguchi (17) published in the Journal of Immunology received the highest number of citations (5,894), followed by Freeman (18) in the Journal of Experimental Medicine (4,514 citations) and Curiel (19) in Nature Medicine (4,090 citations). These landmark studies constitute the theoretical foundation of immune tolerance research in this field. **Figure 6B** displays the top 30 references with the strongest citation bursts since 2000. Most citation bursts were concentrated between 2003 and 2012, with the earliest being the study by Hori (20) published in Science. However, only two publications—Chinen (2016) (21) and Domínguez-Villar (22)—exhibited citation bursts in the past five years, indicating a declining trend in publication activity within the field of peripheral immune tolerance in recent years.

### 3.6 Analysis of journals

**Figure 7:**
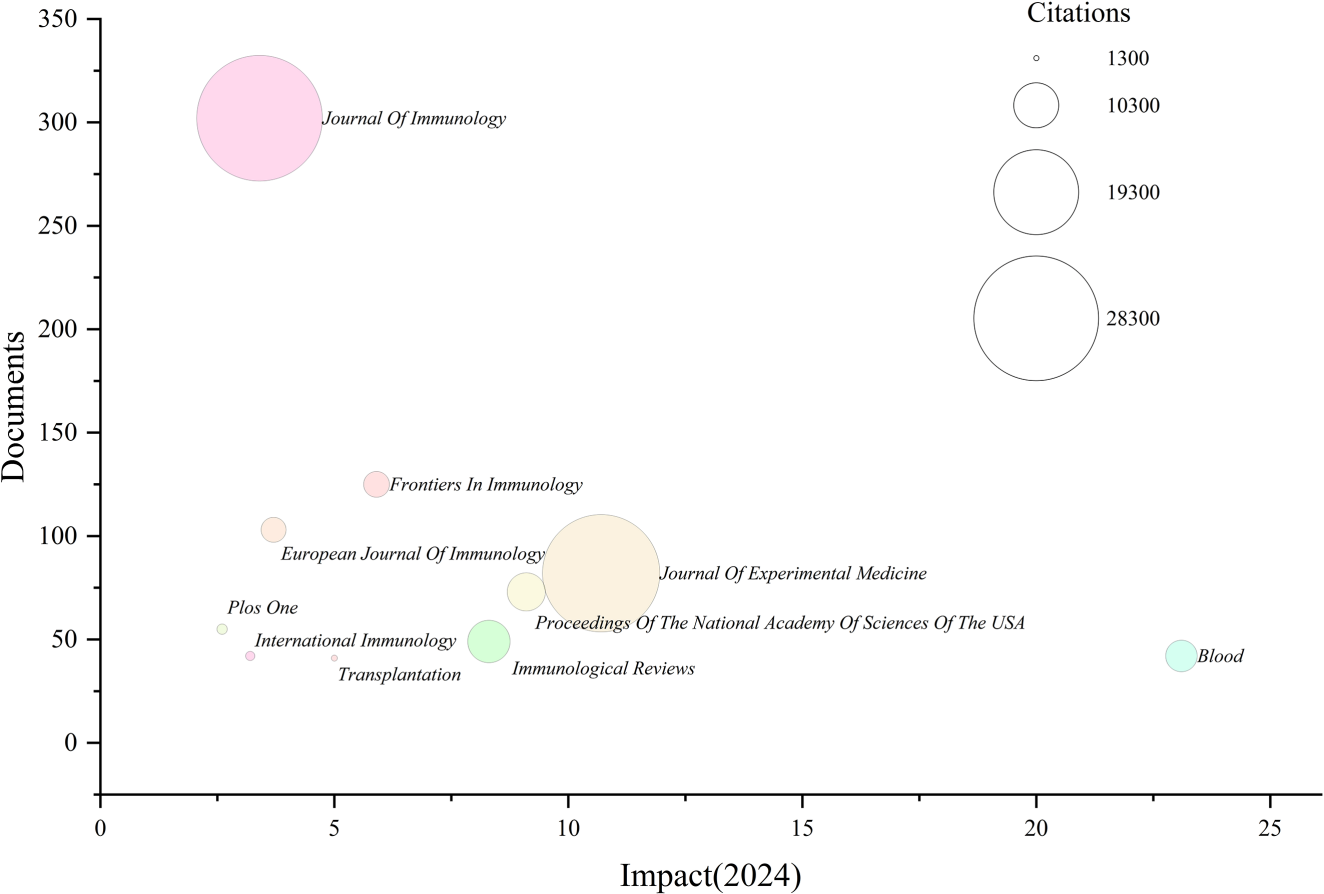
Bubble chart showing the publication output, impact factor, and citation counts of the top 10 journals in peripheral immune tolerance research.

Finally, we performed a statistical analysis of the journals in which the included publications were published. **Figure 7** and **Table 4** summarize the journals contributing most to research on peripheral immune tolerance. **Figure 7** presents a bubble chart, where the bubble size represents the number of citations received by each journal, the x-axis indicates the journal impact factor, and the y-axis shows the number of publications. In theory, journals represented by larger bubbles located toward the upper right corner demonstrate higher overall influence. The Journal of Immunology published the highest number of articles (302), followed by Frontiers in Immunology (125) and the European Journal of Immunology (103). Several journals with moderate publication counts showed high citation levels, such as Journal of Experimental Medicine (82 publications; 26,480 citations) and PNAS (73 publications; 8,626 citations). High-impact journals including Blood (impact factor 23.1), Immunity (26.3), and Nature Immunology (27.6) appear on the plot with relatively lower publication volumes but larger bubble sizes indicating substantial citation influence. Overall, the bubble chart visually aligns with the tabulated data, showing that journals differ considerably in publication output, impact factor, and accumulated citations.

**Table 4:** The top 20 journals by publication count.

Journal	Publications	Citations	Impact(2024)
Journal Of Immunology	302	28375	3.4
Frontiers In Immunology	125	5849	5.9
European Journal Of Immunology	103	5630	3.7
Journal Of Experimental Medicine	82	26480	10.7
Proceedings Of The National Academy Of Sciences Of The USA	73	8626	9.1
Plos One	55	2332	2.6
Immunological Reviews	49	9625	8.3
Blood	42	7151	23.1
International Immunology	42	2063	3.2
Transplantation	41	1337	5
Immunology	35	983	5
Journal Of Clinical Investigation	35	3222	13.6
Clinical And Experimental Immunology	34	1439	4.1
Cellular Immunology	33	1094	2.9
Immunity	31	8596	26.3
Current Opinion In Immunology	29	2900	5.8
Nature Immunology	27	8322	27.6
Journal Of Leukocyte Biology	26	918	3.1
Immunology And Cell Biology	23	1148	3
Trends In Immunology	23	4485	13.9

## 4 Discussion

Clinically, peripheral immune tolerance serves as the biological foundation for both the prevention of autoimmune diseases and the reduction of transplant rejection (23). It is also frequently exploited by the tumor microenvironment to achieve immune evasion (19). Consequently, it has become a key target for immune regulation and translational therapy. In bibliometrics, the results are often subjective and descriptive in nature (24). Therefore, how to ensure that the analysis provides practical and meaningful insights is a critical issue. In this study, data from the Web of Science Core Collection and Scopus databases were retrieved, integrated, and analyzed using multiple bibliometric and visualization tools. A systematic bibliometric analysis was conducted to explore publication trends, keyword evolution, collaborative networks of countries, institutions, and authors, as well as citation patterns and journal distributions in the field of peripheral immune tolerance. This analysis identified the research hotspots, academic core networks, and potential knowledge gaps, providing valuable insights and reference for future studies in project development, mechanistic exploration, targeted drug discovery, autoimmune disease therapy, and scientific knowledge dissemination.

Over the past three decades, the number of publications on peripheral immune tolerance has shown an overall upward trend since the 1990s, reaching its peak around 2008 and gradually declining thereafter. Several external structural factors may have contributed to the decline in publication output after 2008. First, since 2000, the global number of academic journals has increased substantially due to the expansion of open-access and online publishing models. For example, an analysis of Scopus-indexed journals showed that approximately 41.6% of the indexed titles were added between 2000 and 2019, representing more than 11,000 newly indexed journals during this period. Second, changes in the coverage and indexing policies of bibliographic databases may affect longitudinal retrieval trends. Both Scopus and Web of Science regularly revise their content selection criteria and indexing policies, which could partly account for this pattern. Third, over the past decade, the scientific influence of previously dominant immunology journals has gradually declined. For instance, The Journal of Immunology reached a minor peak in 2009 with 1,891 publications, but its annual output sharply dropped to 369 papers by 2024, reflecting a notable contraction in the peripheral immune tolerance literature. Fourth, following the identification of key immune checkpoints such as PD-1/PD-L1 and the rise of cancer immunotherapy, the research focus gradually shifted from fundamental tolerance mechanisms toward tumor immunology and clinical applications (25). For instance, since 2015, PD-1/PD-L1–based immunotherapies have become the standard treatments for multiple malignancies (26). Furthermore, evolutionary therapeutic approaches were also extensively explored during that period, including chimeric antigen receptor regulatory T cells (CAR-Treg) and antigen-specific Treg strategies. These approaches enhance the antigen specificity and stability of Treg cells through genetic engineering, representing an important step in translating fundamental mechanisms of immune tolerance into clinical applications (27, 28). This paradigm shift has inevitably reduced scholarly attention toward the “traditional” field of peripheral immune tolerance, resulting in a gradual decline in both publication output and citation frequency since 2008. Fifth, the declining interest in classical tolerance models—most notably Type 1 diabetes (T1D)—also likely contributes to the apparent reduction in publication counts in recent years. For several decades, T1D was one of the dominant disease models for studying peripheral immune tolerance breakdown, autoreactive T cells and strategies for antigen-specific tolerance induction, particularly through the NOD mouse and early clinical trials. However, more recent work has progressively shifted the T1D field toward broader questions of disease heterogeneity, β-cell stress biology and multi-omics–based endotyping, rather than focusing exclusively on tolerance mechanisms. In their comprehensive Lancet Seminar, Atkinson and colleagues emphasized that recent advances have largely concentrated on genetic risk, environmental modifiers, pancreatic pathology and improved clinical management, while immune tolerance–directed interventions represent only one component within a much wider pathogenic framework (29). At the same time, translational efforts in T1D have become increasingly focused on specific interventional strategies, such as adoptive transfer of regulatory T cells, rather than on classical tolerance models per se. For example, Bluestone and colleagues reported a phase 1 trial in which *ex vivo*–expanded autologous polyclonal Tregs were infused into patients with recent-onset T1D, demonstrating safety and persistence of the transferred cells, and representing a concrete step toward cell-based immunotherapy (16). Taken together, these trends suggest that classical models like T1D, while still important, no longer occupy the same central position as “prototypic tolerance models” in the literature. This thematic shift—from disease-specific tolerance models to broader questions of immune regulation, tissue microenvironments and targeted interventions—likely contributes to the observed decline in publications explicitly tagged with traditional tolerance-model keywords.

It is worth noting that Sakaguchi proposed the concept of regulatory T cells (Tregs) and demonstrated their pivotal role in immune tolerance in 1995 (17), which led to a rapid surge in related research. For this groundbreaking discovery, Shimon Sakaguchi, together with Fred Ramsdell and Mary E. Brunkow, was awarded the 2025 Nobel Prize in Physiology or Medicine. With the 2025 Nobel Prize recognizing seminal contributions to this area, research interest in peripheral immune tolerance is expected to experience renewed growth in the coming years.

Through the analysis of large-scale keyword data, it is evident that “autoimmunity” and “Treg/FOXP3” have long remained the central research foci in the field of immune tolerance. This is closely related to the mechanism by which failure of peripheral immune tolerance directly triggers autoimmune diseases: once tolerance is lost, self-reactive effector cells can mediate inflammatory immune responses (30). The FOXP3 gene functions as the master regulatory switch of Treg cells, controlling their development and function, thereby maintaining immune tolerance (20). This factor reprograms CD4 by interacting with co factors such as NFAT and Runx1 to enhance T cell activity, thereby increasing the expression of inhibitory molecules including CD25 and CTLA-4, while inhibiting effector genes such as IL2 and IFNG (31) FOXP3 suppresses excessive immune responses, prevents the onset of autoimmune diseases, and also plays critical roles in tumor immunity and transplantation tolerance. Mutations in this gene can lead to severe autoimmune disorders such as IPEX syndrome (32, 33). Consistent with these mechanistic foundations, accumulating clinical evidence further underscores that disruptions in Treg number and function represent a common pathway driving diverse immune-mediated diseases. In vitiligo, two recent meta-analyses demonstrated significantly reduced Treg frequency, impaired suppression of CD4^+^ and CD8^+^ T cells, and decreased expression of FOXP3, IL-10, and TGF-β in both skin and blood, with disease activity correlating with the magnitude of Treg dysfunction (34, 35). Importantly, therapeutic interventions capable of restoring Treg function—such as narrow-band UVB or Treg-modulating treatments—were associated with increases in Treg frequency and FOXP3 expression, highlighting the translational relevance of tolerance restoration. Beyond autoimmunity, FOXP3^+^ Tregs also exert context-dependent effects in cancer. A systematic review of 76 studies showed that high intratumoral Treg infiltration was generally associated with poorer overall survival in many solid tumors, whereas it correlated with improved outcomes in certain cancers such as colorectal, head and neck, and esophageal malignancies (36). Together, these findings illustrate that Treg dysregulation—whether through numerical deficiency, impaired suppressive capacity, or altered tissue localization—serves as a unifying immunological mechanism across autoimmune disease and cancer, reinforcing the centrality of FOXP3-mediated pathways in shaping peripheral immune tolerance.

As shown in **Figure 4**, recent trending topics emphasize how peripheral immune tolerance is increasingly being investigated within specific disease and therapeutic contexts rather than under traditional mechanistic labels. “Systemic lupus erythematosus (SLE)” represents a prototypical systemic autoimmune disorder in which the breakdown of peripheral immune tolerance to nuclear self-antigens leads to autoantibody production and multi-organ damage. Current therapeutic strategies, including B cell–targeted agents and emerging Treg-based approaches, are partly aimed at restoring defective peripheral immune tolerance (37). “Treg cells” and “FOXP3” denote the core cellular and transcriptional regulators responsible for enhancing peripheral immune tolerance, as discussed earlier. The keyword “tumor microenvironment” reflects growing interest in how tumors exploit peripheral immune tolerance mechanisms: within this milieu, Treg cells can be recruited and accumulate abundantly, acting as one of the major barriers to effective antitumor immune responses (38). PD-1 is an inhibitory receptor expressed on activated T and B cells, playing a critical role in regulating peripheral immune tolerance (18). The interaction between PD-1 and its ligand PD-L1 suppresses T-cell activation and enhances tumor immune tolerance (39).The emergence of “immune checkpoint inhibitors” and “PD-1/PD-L1” indicates a complementary trend, wherein peripheral immune tolerance pathways are intentionally disrupted to potentiate anti-tumor immune responses. This highlights that the same checkpoints maintaining self-tolerance can serve as therapeutic targets in cancer immunotherapy (40). Consequently, research on PD-1–related tumor immune pathways has increased in recent years, making PD-1/PD-L1 one of the most frequently appearing and intensively studied keywords within the domain of peripheral immune tolerance. Finally, the appearance of “nanoparticles” underscores the rapid advancement of biomaterial-based delivery systems designed to promote antigen-specific peripheral immune tolerance in autoimmune diseases and transplantation, offering promising avenues for novel targeted therapeutics (41).

Collectively, these emerging keywords suggest that although the explicit use of traditional terminology has declined, research on peripheral immune tolerance is being restructured around specific clinical contexts and intervention strategies, reflecting an evolution from mechanistic studies toward translational and application-oriented paradigms.

National research output reflects the development status of a country’s scientific capacity. Bibliometric analysis helps compare research input–output efficiency across countries and identify patterns of international collaboration (42, 43). In the field of peripheral immune tolerance, countries such as the United States, Germany, and China have contributed the majority of research outputs. The United States has maintained a dominant position, leading with 1,351 publications and 143,081 citations, thereby exerting a strong influence on research directions. It also serves as the central hub in the international collaboration network, exhibiting the highest collaboration strength. The institution with the largest number of publications is Harvard University, with 90 papers and the highest citation count (10,768), reflecting its core leadership in this field. The most productive author is Roncarolo Maria Grazia, who has published 23 papers with 9,801 citations. **Tables 1**, **3** provide valuable references for identifying potential collaborating institutions and authors for future research. However, a higher publication count does not necessarily indicate a greater likelihood of future collaboration. Several highly productive authors were most active in earlier decades and are no longer contributing to current research trends. For example, although Weiner, Howard L. has accumulated a substantial citation count (6,548 citations), his active involvement in the field of peripheral immune tolerance largely ceased around 2010, suggesting that his research focus has shifted to other areas. Consequently, identifying meaningful collaboration opportunities requires prioritizing authors based not only on overall influence but also on their recent years of activity. Modern bibliometric platforms support such refined assessments; for instance, the Web of Science Core Collection allows users to query authors by ORCID, enabling the retrieval of both their high-impact publications and their most recent output. These tools help investigators more accurately identify active and influential collaborators who are currently shaping the field. To sum up, such cross-national collaboration facilitates resource sharing and knowledge exchange, and is expected to further advance research on immune tolerance toward deeper mechanistic insights and translational applications.

In addition, an in-depth analysis was performed on the highly cited and co-cited publications in the field of peripheral immune tolerance. The most frequently cited publication was the landmark paper by Sakaguchi (17) in the Journal of Immunology, which was the first to systematically demonstrate that regulatory T cells (Tregs) play a critical role in maintaining peripheral immune tolerance and preventing autoimmune responses. This study laid the foundational framework for the Treg concept (17). Subsequent highly cited studies—most notably those by Freeman (18) and Curiel (19)—further established the theoretical and methodological basis of this field. These seminal works defined the immunosuppressive functions of the Treg lineage, elucidated the regulatory mechanisms of the immune checkpoint molecule PD-1/PD-L1, and revealed the core pathways of immune evasion within the tumor microenvironment (18, 19). The most recent co-citation burst was observed for the review by Domínguez-Villar (22) published in *Nature Immunology*, which provided a comprehensive summary of the roles and dysregulation mechanisms of Treg cells in autoimmune diseases (22).

In **Figure 6**, two publications stand out as being both highly cited and highly co-cited: *Steinman RM, 2003, Annu. Rev. Immunol.* and *Vignali DAA, 2008, Nat. Rev. Immunol.* The former discusses the role of dendritic cells (DCs) in the induction of immune tolerance (44), while the latter provides a systematic review of the mechanisms underlying Treg-mediated suppression, highlighting their context-dependent and complementary nature (45). Together, these landmark studies represent the intellectual backbone of peripheral immune tolerance research and serve as essential references for future investigators seeking to advance understanding of immune regulation, tolerance induction, and translational immunotherapy.

The major research findings in the field of peripheral immune tolerance have predominantly been published in specialized immunology journals. The Journal of Immunology is the most prolific journal, with 302 publications and a total of 28,375 citations. The highest impact factor is observed for Nature Immunology, with an impact factor of 27.6 in 2025. Within tolerance-related research, Frontiers in Immunology has emerged as one of the most popular and active publication platforms. By examining journal publication volume, citation frequency, and impact indicators, we visualized these results in **Figure 7**, providing robust data support for future researchers to identify suitable journals for dissemination and to facilitate the continuous development and advancement of research on peripheral immune tolerance.

It is worth noting that Gao et al. conducted a bibliometric analysis of global research trends on regulatory T cells in neurological diseases and found that the United States was the most productive country, followed by China. Frontiers in Immunology was identified as the leading publication venue in this field, and keywords such as ‘regulatory T cells,’ ‘autoimmunity,’ and ‘inflammation’ were among the most frequently used author keywords (46). These findings are largely consistent with our results, further highlighting the dominant contributions of the United States and China in Treg-related research, as well as the central role of terms like Treg/FOXP3 and autoimmunity in global research hotspots.

Several limitations should be acknowledged in this study. First, all data were retrieved exclusively from the Web of Science Core Collection and Scopus databases. Although these two sources represent the most authoritative repositories for bibliometric analysis, they do not encompass all relevant publications—particularly book chapters and conference proceedings. In addition, PubMed was excluded due to its incomplete citation metadata and lower compatibility with bibliometric tools compared with Web of Science and Scopus. Including PubMed data might introduce considerable bias to the overall analysis, although some valuable insights may be missed as a result. Second, bibliometric analysis primarily emphasizes quantitative indicators—such as publication counts, citation frequencies, and co-occurrence strength—which may not fully capture the qualitative scientific impact or innovation of individual studies. For instance, recent high-quality publications that have not yet accumulated sufficient citations may be underrepresented. Therefore, continuous updates and integration of dynamic citation data will be necessary in future studies to ensure that bibliometric assessments remain accurate and reflective of current research trends.

## Data Availability

The original contributions presented in the study are included in the article/**Supplementary Material**. Further inquiries can be directed to the corresponding author.
